# Response to letter: More fat, less migration: breast density as a predictor of sentinel lymph node non-visualization in breast cancer

**DOI:** 10.1186/s13550-021-00853-z

**Published:** 2021-10-29

**Authors:** Youssef Chahid, Hein J. Verberne, Jan Booij

**Affiliations:** Amsterdam UMC Locatie AMC, Amsterdam, Noord-Holland The Netherlands

## To the Editor,

We would like to thank Quak and colleagues for their interest in our recent publication where we found that age ≥ 70 years, body mass index (BMI) ≥ 30 kg/m^2^ and nonpalpable tumors were independent risk factors for sentinel lymph node (SLN) nonvisualization during preoperative lymphoscintigraphy [1].

We read with great interest the findings of Quak et al. [2] that age and BMI are significantly associated with breast density. Patients with high fat content in breast tissue (i.e., low density) were, based on univariate analysis, significantly more at risk of SLN nonvisualization compared to patients with less fatty breast densities. Based on the combined findings of our study and that of Quak et al., it seems that SLN nonvisualization is driven in part by BMI and thereby lower breast density (more fatty breast tissue). Unfortunately, due to the lack of a multivariable analysis, we cannot properly assess the possible influence of breast density in relation to other risk factors (i.e., age and BMI) for SLN nonvisualization.

Nevertheless, based on the findings mentioned above, we do agree that breast density should be further investigated as a risk factor for SLN nonvisualization to optimize the lymphoscintigraphy protocol.

Yours sincerely,

Youssef Chahid, Hein Verberne, Jan Booij.
